# Detection of doublecortin domain-containing 2 (*DCDC2*), a new candidate tumor suppressor gene of hepatocellular carcinoma, by triple combination array analysis

**DOI:** 10.1186/1756-9966-32-65

**Published:** 2013-09-14

**Authors:** Yoshikuni Inokawa, Shuji Nomoto, Mitsuhiro Hishida, Masamichi Hayashi, Mitsuro Kanda, Yoko Nishikawa, Shin Takeda, Hiroyuki Sugimoto, Tsutomu Fujii, Suguru Yamada, Yasuhiro Kodera

**Affiliations:** 1 Gastroenterological Surgery, Nagoya University Graduate School of Medicine, Japan, 65 Tsurumai-cho, Showa-ku, Nagoya 466-8550, Japan

**Keywords:** DCDC2, Hepatocellular carcinoma, Methylation, Triple combination array

## Abstract

**Background:**

To detect genes correlated with hepatocellular carcinoma (HCC), we developed a triple combination array consisting of methylation array, gene expression array and single nucleotide polymorphism (SNP) array analysis.

**Methods:**

A surgical specimen obtained from a 68-year-old female HCC patient was analyzed by triple combination array, which identified doublecortin domain-containing 2 (*DCDC2*) as a candidate tumor suppressor gene of HCC. Subsequently, samples from 48 HCC patients were evaluated for their *DCDC2* methylation and expression status using methylation specific PCR (MSP) and semi-quantitative reverse transcriptase (RT) PCR, respectively. Then, we investigated the relationship between clinicopathological factors and methylation status of *DCDC2*.

**Results:**

*DCDC2* was revealed to be hypermethylated (methylation value 0.846, range 0–1.0) in cancer tissue, compared with adjacent normal tissue (0.212) by methylation array in the 68-year-old female patient. Expression array showed decreased expression of *DCDC2* in cancerous tissue. SNP array showed that the copy number of chromosome 6p22.1, in which *DCDC2* resides, was normal. MSP revealed hypermethylation of the promoter region of *DCDC2* in 41 of the tumor samples. *DCDC2* expression was significantly decreased in the cases with methylation (*P* = 0.048). Furthermore, the methylated cases revealed worse prognosis for overall survival than unmethylated cases (*P* = 0.048).

**Conclusions:**

The present study indicates that triple combination array is an effective method to detect novel genes related to HCC. We propose that *DCDC2* is a tumor suppressor gene of HCC.

## Background

Hepatocellular carcinoma (HCC) is currently the fifth most common malignancy worldwide [1], and its overall incidence is steadily rising. In spite of the therapeutic options for HCC such as hepatic resection [2], radiofrequency ablation [3], transcatheter arterial chemoembolization [4], and sorafenib [5], the prognosis of patients with advanced HCC remains poor [6,7]. Therefore, research to clarify the mechanisms of hepatocarcinogenesis is urgently required [8].

Gene expression microarray analysis has revealed many cancer-related genes in HCC [9]. This method enables the expression status of all genes to be investigated simultaneously [10]. Furthermore, single nucleotide polymorphism (SNP) arrays have made it possible to detect copy number changes as well as copy-neutral loss of heterozygosity (LOH) [11]. Recently we developed a double combination array analysis consisting of gene expression array and SNP array analysis, and reported a number of tumor suppressor genes in HCC [12-17]. In these studies, we hypothesized that DNA methylation of the promoter region of these genes downregulated gene expression, causing HCC progression. In addition to this double combination array analysis, we obtained further data from the same specimens using methylation array analysis to make this association of DNA methylation more conclusive. We named it triple combination array analysis; this method seems to be an efficient procedure for the detection of tumor suppressor genes of HCC [18].

Doublecortin domain-containing 2 (*DCDC2*) is a candidate tumor suppressor gene detected by this triple combination array analysis. This gene encodes a member of the doublecortin family [19], and contains two doublecortin domains. The doublecortin domain has been demonstrated to bind tubulin and enhance microtubule polymerization [19,20], and mutations in this gene have been associated with dyslexia [21-24]. However, there are only a few reports of the relationship between *DCDC2* and cancer [25]. In addition, no previous study has researched the role of *DCDC2* in HCC. Although it had been considered that *DCDC2* gene had an impotrtant role in neuroendocrine systems, the expression of the gene was reported in GeneCards relatively strongest in liver in whole human organs including brain. Therefore, we selected this gene for this study, because we predicted the gene might have some role in liver.

## Methods

### Sample collection and DNA preparation

Nine HCC cell lines (HepG2, Hep3B, HLE, HLF, HuH1, HuH2, HuH7, PLC/PRF-5 and SK-Hep1) were obtained from the American Type Culture Collection (Manassas, VA, USA). The cell lines were cultured in RPMI-1640 supplemented with 10% fetal bovine serum, and incubated in 5% CO_2_ at 37°C.

A 68-year-old woman with chronic hepatitis C was diagnosed with HCC in the right lobe and underwent liver resection. Specimens of her tumor and adjacent non-tumorous tissues were excised, and total RNA and DNA were extracted. Total RNA was sent to the manufacturer of Affymetrix to prepare it for expression array analysis. Genomic DNA was used for a SNP-Chip array, and bisulfite-converted DNA was used for the Ilumina Infinium HumanMethylation 27 BeadChip (Illumina, San Diego, CA, USA). The tumor was pathologically confirmed as HCC. RNA and DNA of tumor samples were extracted from an area consisting of >80% cancerous cells.

HCC tissue (HTs) and normal tissue (NTs) samples were obtained from 48 patients (43 males, five females) who underwent liver resection at Nagoya University Hospital, Nagoya, Japan between 1994 and 2001. The patients were aged from 39 to 77 years (mean ± SD, 62.4 ± 7.9 years). Thirty-eight patients had hepatitis C and seven had hepatitis B. The median duration of follow-up was 80.7 months (range 15.2–213.1 months). All tissues were reviewed pathologically to confirm the diagnosis of HCC. Written informed consent, as required by the institutional review board, was obtained from all patients. The tissue samples were immediately frozen in liquid nitrogen and stored at −80°C until required. Genomic DNA was obtained from the tissue samples by proteinase K digestion, followed by phenol/chloroform extraction.

### RNA isolation, microarray and gene chip affymetrix procedures

The expression array and SNP array were performed, as previously described [12-17], using total RNA and DNA extracted from the 68-year-old woman’s tissue samples.

### Methylation array platform

The Illumina Infinium HumanMethylation27 BeadChip protocol requires 500 ng to 1 μg of bisulfite-converted DNA [26]. Of the approximately 28 million CpG sites found throughout the haploid human genome, Illumina initially designed Infinium methylation probes for 27,578 CpG sites located in promoter regions (up to 1 kb upstream or 500 bp downstream of the transcription start sites). Of these, 27,324 CpG sites relate to 14,475 consensus coding sequences, including around 1000 cancer-associated genes, and 254 CpG sites relate to approximately 100 micro-RNA genes. The probes were preferentially selected to occur within CpG islands using the NCBI “relaxed” definition of a CpG island: CpG islands identified bioinformatically with a CpG content of >50% and an observed/expected ratio of >0.6 [27].

Bisulfite-converted DNA is then whole-genome amplified, enzymatically fragmented, and hybridized to the array. During hybridization, the bisulfite-converted DNA anneals to methylation-specific probes on the chip. Each CpG locus is represented by two bead types, one of which is specific to the methylated state and the other is specific to the unmethylated state, which is directly related to the underlying sequence change catalyzed during bisulfite conversion. Therefore, for each CpG site, a possible C/T variant can be assayed through the single-base extension step, which is possible because of the ability to hybridize to either the “protected” methylated cytosine or the converted (unmethylated) thymine.

After hybridization, a single-base extension step is carried out using a multi-layer staining process, as described below. The BeadChip is then scanned on the Illumina iScan and the resulting “idat” files are analyzed using BeadStudio software. The output of the BeadStudio analysis is a β-value for each CpG site. This is a continuous value between 0 and 1 where 0 = 0% methylation and 1 = 100% methylation at a given CpG site. Therefore, this assay enables quantitative analysis of methylation at individual CpG sites.

### Reverse transcription-polymerase chain reaction (RT-PCR)

*DCDC2* mRNA expression was analyzed by semi-quantitative RT-PCR and real-time RT-PCR. Total RNA (10 μg) isolated from nine HCC cell lines, primary HTs and NTs were used to generate cDNAs. The resulting cDNAs were then amplified by PCR primers for *DCDC2* (sense, 5′- GCT TCA GGA GCC GTG CAC TA -3′ in exon 4); antisense 5′- CCC CGC TCC TCA GAG TGA TT -3′ in exon 5), which amplified a 146-bp product. Initial denaturation at 94°C for 5 min was followed by amplification consisting of 35 cycles of 94°C for 10 s, 60°C for 8 s, and 72°C for 6 s. RT-PCR of beta-actin was performed to confirm equal amounts of cDNA was used as templates. Each PCR product was loaded directly onto 3% agarose gels, stained with ethidium bromide, and visualized under UV illumination.

### Real-time quantitative RT-PCR analysis

PCR was performed with the SYBR Green PCR Core Reagents kit (Perkin-Elmer Applied Biosystems, Foster City, CA, USA) under the following conditions: 1 cycle at 95°C for 10 s, followed by 40 cycles at 95°C for 5 s and at 60°C for 30 s. SYBR Green emission was detected in real-time with an ABI prism 7000 Sequence Detector (Perkin-Elmer Applied Biosystems). The primers used in PCR were the same as those described above for RT-PCR. For standardization, the expression of *GAPDH* was quantified in each sample. Quantitative RT-PCR was performed at least three times, including negative controls without template. The expression of *DCDC2* was normalized for that of *GAPDH* in each sample.

### Methylation-specific PCR (MSP)

DNA from HCC cell lines, HTs and NTs were subjected to bisulfite treatment. Briefly, 2 μg of DNA was denatured by NaOH and modified by sodium bisulfite. DNA samples were then purified using the Wizard purification resin (Promega Corp., Madison, WI, USA), treated with NaOH, precipitated with ethanol, and resuspended in water. Primer pairs were used to detect methylation (sense, 5′- AGG TCG TTG GGA TAG CGG AG -3′; antisense, 5′- TCA TCT TCC CCG CTA ACC GC -3′; 73-bp product) and non-methylation (sense, 5′- GGG TGT GGT GAG GTT GTT GG -3′; antisense, 5′- CTT CCC CAC TAA CCA CCA CC -3′; 79-bp product) of the *DCDC2* promoter region near exon 1. The MSP and unmethylated-specific PCR (UNMSP) amplification consisted of denaturation at 94°C for 5 min followed by 35 cycles at 94°C for 8 s, 60°C for 5 s, and 72°C for 3 s. The PCR products were loaded directly onto 3% agarose gels, stained with ethidium bromide, and visualized under UV illumination.

### Sequence analysis

Bisulfite-treated genomic DNA obtained from HCC cell lines was sequenced and PCR was performed in all cases. We performed semi-nested PCR to gain adequate products for TA cloning. PCR amplification consisted of denaturation at 94°C for 3 min followed by 35 cycles of 94°C for 10 s, 52°C for 10 s and 72°C for 20 s with primer pairs (sense 5′- TTT AGT GTT TTT TTT GGG TG -3′; antisense, 5′ - CTA AAC ACC TTC TTC TCA TG -3′ ; 312-bp product). The products were used as templates of subsequent PCRs with primer pairs consisting of the same sense, and different antisense (antisense, 5′- AAC AAA TAA CTA AAC CTA AC -3′; 219-bp product). The PCR products were subcloned into a TA cloning vector (Invitrogen, Carlsbad, CA, USA). Six cloning samples were picked out from two HCC cell lines (HuH2 and SK-Hep1). Each DNA clone was mixed with 3 μl of the specific primer (M13) and 4 μl of Cycle Sequence Mix (ABI PRISM Terminator v1. 1 Cycle Sequencing Kit; Applied Biosystems, Foster City, CA, USA). Samples were then subjected to the following cycling conditions: 95°C for 30 s followed by 25 cycles of 96°C for 10 s, 50°C for 5 s, and 60°C for 4 min, and then purified by ethanol precipitation. Sequence analysis was carried out using an Applied Biosystems ABI310, and sequence electropherograms were generated using ABI Sequence Analysis software version 3.0.

### 5-Aza-2′-deoxycytidine (5-aza-dC) treatment

To confirm that promoter hypermethylation was responsible for silencing of gene expression, the nine HCC cell lines were treated with 1 μM 5-aza-dC (Sigma-Aldrich, St. Louis, MO, USA) to inhibit DNA methylation. Cells (1.5 × 10^6^) were cultured for 6 days with medium changes on days 1, 3, and 5. On day 6, the cells were harvested, RNA was extracted, and RT-PCR was performed as described above.

### Western blotting analysis

Cultured cells were washed twice with phosphate-buffered saline and lysed by lithium dodecyl sulfate (LDS) buffer (Invitrogen). Protein lysates were resolved on 10% SDS polyacrylamide gel, electrotransferred to polyvinylidene fluoride membranes using iBlot Gel Transfer Device (Invitrogen) and blocked in 5% nonfat dry milk. Membranes were immunoblotted overnight at 4°C with a rabbit anti-DCDC2 antibody (ab106283; Abcam plc, Cambridge, UK) followed by peroxidase-conjugated secondary antibodies. As a control, a mouse monoclonal anti-beta-actin antibody (Abcam plc,) was used. Signals were detected by enhanced chemiluminescence (Lumivision PRO HSII, Aisin Seiki Co., LTD, Kariya, Japan).

### Immunohistochemistry

We investigated DCDC2 protein expression by immunohistochemistry in the same samples analyzed by the arrays and in 38 HCCs whose samples were available in a well-preserved condition. Cut sections were prepared from formalin-fixed and paraffin-embedded tissues for DCDC2 staining. Samples were treated with 3% H_2_O_2_ to inhibit endogenous peroxidase, and then subjected to antigen retrieval using 10 mM citrate buffer five times at 95°C for 10 min. Sections were incubated with Histofine SAB-PO(R) (Nichirei, Tokyo, Japan) for 10 min, to limit non-specific reactivity, and then incubated with DCDC2 antibody produced in rabbit (ab106283; Abcam plc) diluted 1:2000 in ChemMatet antibody diluent (Dako) for 12 h. All stains were developed for 15 min using liquid diaminobenzidine (DAB) as the substrate (Nichirei). We determined staining properties setting vessels as integral control, and made a comparison of DCDC2 expression between HCC tissues and corresponding non-cancerous tissues. To avoid being subjective, specimens were randomized and coded before analysis, which was conducted by two independent observers, who evaluated all specimens at least twice within a given interval to minimize intra-observer variation.

### Statistical analysis

Continuous variables are expressed as medians (range) and comparisons were made using the Mann Whitney U test. Categorical variables were compared using χ^2^ tests or Fisher’s exact tests, where appropriate. Overall survival rates were analyzed by Kaplan-Meier and log-rank tests. All statistical analyses were performed using JMP software version 9.0.2 (SAS International Inc., Cary, NC, USA). The level of statistical significance was set at *P* < 0.05.

## Results

### Results of expression, SNP, and methylation-arrays

To identify novel tumor–related genes in HCC, we first searched for genes with decreased expression in HCC samples compared with corresponding normal tissue. According to the expression array results, *DCDC2* was strongly downregulated in HCC tissue. The decreased values (log 2 ratio) were −2.2 in a point of the expression array chip (Table 1).

**Table 1:** Expression array analysis of the 68-year-old female’s surgical HCC sample

**Probe set ID**	**Gene symbol**	**Log2 ratio**	**Sample**	**Signal**	**Detection**
222925_at	DCDC2	**−2.2**	Normal	148.3	P
			Tumor	36.9	P

*HCC* hepatocellular carcinoma, *DCDC2* doublecortin domain-containing 2.

**Figure 1:**
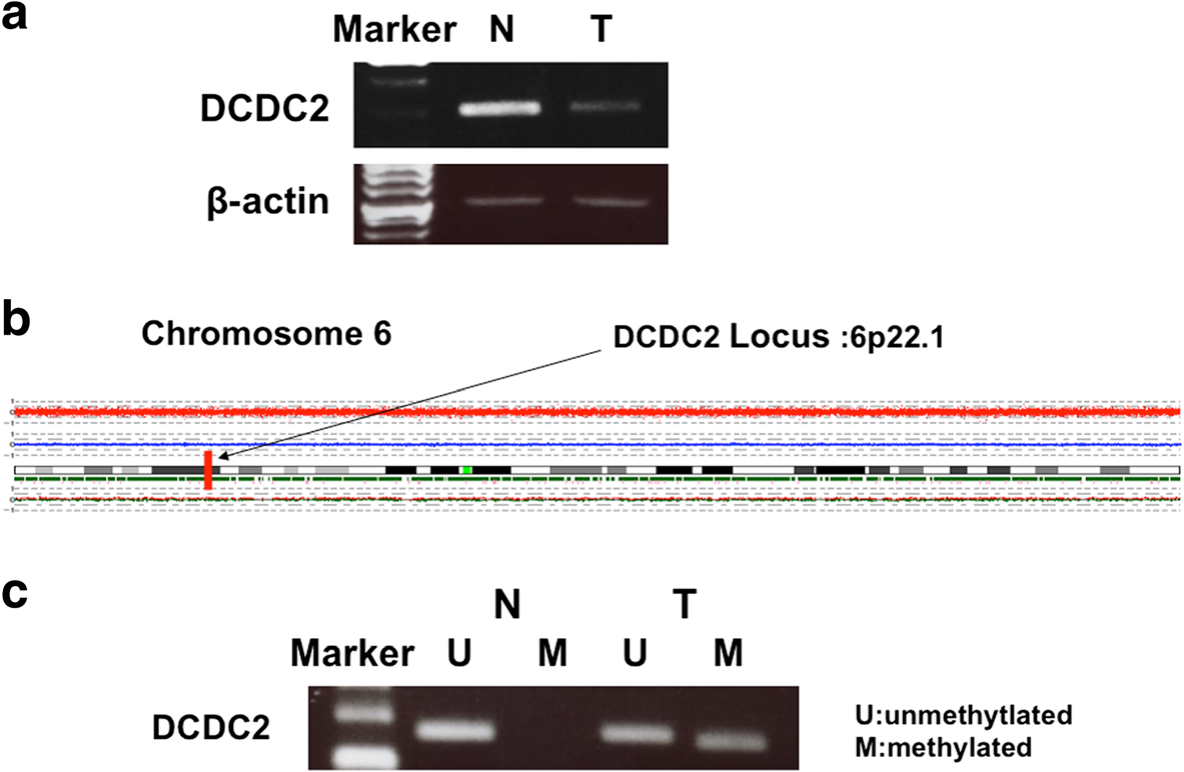
**Results of experiments from a specimen from a 68-year-old woman. (a)** Semi-quantitative RT-PCR showed downregulation of *DCDC2* in the tumor sample compared with corresponding normal tissue. **(b)** Copy number analysis of chromosome 6 by SNP array in the HCC sample did not show any deletion or amplification on 6p22.1, the *DCDC2* locus. **(c)** MSP showed promoter hypermethylation in the tumor sample alone.

We confirmed reduced expression of *DCDC2* mRNA in tumor tissue by semi-quantitative RT-PCR in the case whose samples were used for the array analysis (Figure 1a).

Next, we checked the results of the SNP array. We observed deletions in 3q, 8p, 11q, 12p, 12q, 16p, 17p, 19p, and X chromosomes, and chromosomal gain in 1q, 3q, 11q, 12p, and 12q. The copy number of chromosome 6, which contains *DCDC2*, did not show any deletions and amplifications (Figure 1b). Also, we looked for detailed data of the SNP array at the *DCDC2* gene locus at 6p22.1, and found 29 SNPs. Twelve of these 29 SNPs showed a heterozygous AB allele in both the non-cancerous and cancerous samples (Table 2). These results suggest that the *DCDC2* gene locus retained biallelically.

**Table 2:** Results of SNP signal at the DCDC2 gene locus

**Probe set ID**	**Chromosome**	**Physical position**	**Normal call**	**Confidence**	**Tumor call**	**Confidence**
SNP_A-2175183	6	24175005	AB	0.007813	AB	0.023438
SNP_A-1934540	6	24175527	AB	0.007813	AB	0.007813
SNP_A-2079666	6	24202016	AB	0.015625	AB	0.015625
SNP_A-1920269	6	24202874	AB	0.0625	AB	0.132813
SNP_A-2242966	6	24227520	AB	0.007813	AB	0.007813
SNP_A-1825242	6	24238542	AB	0.023438	AB	0.0625
SNP_A-4233820	6	24241681	AB	0.125	AB	0.0625
SNP_A-2042383	6	24317865	AB	0.023438	AB	0.007813
SNP_A-2136345	6	24330431	AB	0.007813	AB	0.007813
SNP_A-4215128	6	24330575	AB	0.015625	AB	0.132813
SNP_A-4242164	6	24353402	AB	0.047363	AB	0.02832
SNP_A-1870108	6	24356599	AB	0.0625	AB	0.039063

*SNP* single nucleotide polymorphism, *DCDC2* doublecortin domain-containing 2.

We subsequently checked the results of the methylation array: the continuous β-values were 0.846 for tumor tissue versus 0.212 for normal tissue, indicating high methylation in HCC sample (Table 3). Using MSP, we confirmed hypermethylation in this gene in the tumor tissue obtained from the 68-year-old woman whose DNA was used for the methylation array (Figure 1c). These results implied that *DCDC2* expression decreased without LOH, possibly because of hypermethylation at the promoter region.

**Table 3:** Methylation array analysis of the 68-year-old female’s surgical HCC sample

**Probe ID**	**Gene symbol**	**Sample**	**Methylation value(0–1)**	**Status**			**Confidence**	**Chromosomal location**
				**Total**	**Unmethylated**	**Methylated**		
cg 16306115	DCDC2	Normal	**0.212**	7096	5569	1527	3.68E-38	Chr6p22.1
		Tumor	**0.846**	9684	1399	8285	3.68E-38	

*HCC* hepatocellular carcinoma, *DCDC2* doublecortin domain-containing 2.

### Effects of inhibiting methylation on DCDC2 expression in nine HCC cell lines

**Figure 2:**
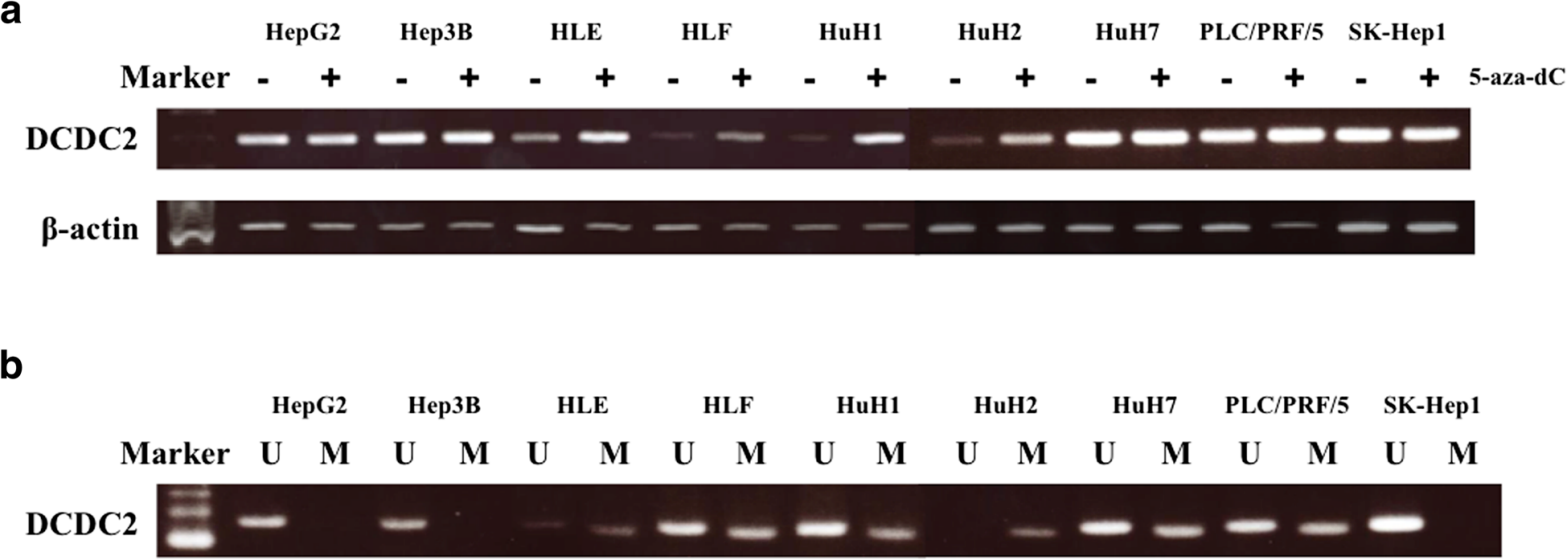
**Results of Semi-quantitative RT-PCR and MSP in nine HCC cell lines. (a)** Semi-quantitative RT-PCR showed reactivation of *DCDC2* expression in five (HLE, HLF, HuH1, HuH2 and PLC/PRF/5) of nine HCC cell lines. **(b)** MSP showed complete methylation in HuH2, partial methylation in HLE, HLF, HuH1, HuH7 and PLC/PRF/5, and no methylation in HepG2, Hep3B and SK-Hep1.

To confirm that promoter hypermethylation led to silencing of *DCDC2* expression, we checked the mRNA expression of the gene before and after 5-aza-dC treatment of nine HCC cell lines. The expression of *DCDC2* in five of these lines, HLE, HLF, HuH1, HuH2 and PLC/PRF/5, was clearly reactivated by 5-aza-dC treatment, as shown by semi-quantitative RT-PCR (Figure 2a).

### MSP and UNMSP of nine HCC cell lines and one HCC case

Then, we designed primers for methylation-specific (MSP) and non-methylation-specific PCR (UNMSP) and checked the methylation status of nine HCC cell lines. For MSP, we obtained bands of appropriate size in lanes containing HLE, HLF, HuH1, HuH2, HuH7, PLC/PRF/5 samples, while in UNMSP, appropriate bands were identified in lanes for all cell lines except HuH2 (Figure 2b). We subsequently identified complete methylation in HuH2, partial methylation in HLE, HLF, HuH1, HuH7 and PLC/PRF/5, and no methylation in HepG2, Hep3B and SK-Hep1.

### Sequence analysis

**Figure 3:**
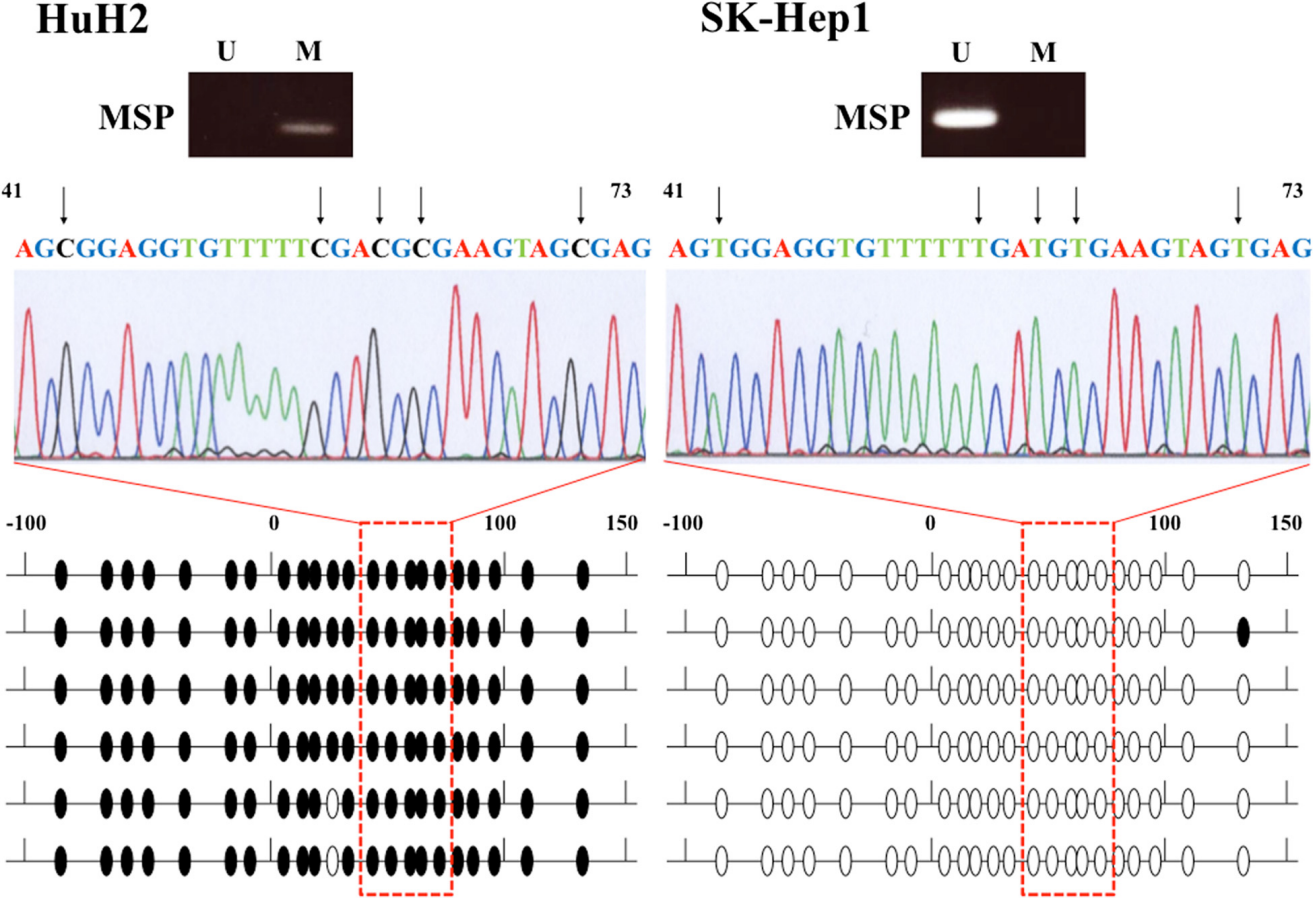
**Sequence analysis of bisulfate-treated DNA in the *DCDC2 *promoter region.** Methylation status of the 22 CpG islands in the six clones by TA cloning method between −100 and +150 from the transcription initiation site of *DCDC2* exon 1 is shown. Closed circles represent methylated CpG islands; open circles indicate unmethylated CpG islands. The CpG islands in the promoter region in HuH2 cells were abundantly methylated, whereas CpG islands in SK-Hep1 cells were abundantly unmethylated. The middle figures in the sequence analysis show the results at the CpG islands between 41 and 73 corresponding to the boxes of the lower figure. The Cs indicate methylated CpG islands. The Ts were converted from C by bisulfite treatment, and indicate unmethylated CpG islands. These results verified the accuracy of MSP and UNMSP in upper figures.

To confirm that MSP amplification was correctly performed, we executed sequence analysis of the *DCDC2* promoter region in HuH2 and SK-Hep1 cells. Almost all CpG dinucleotides in HuH2 were methylated, while those of SK-Hep1 were unmethylated (Figure 3). These results verified the accuracy of MSP and UNMSP.

### MSP and UNMSP of normal and tumor tissues from 48 HCC patients

**Figure 4:**
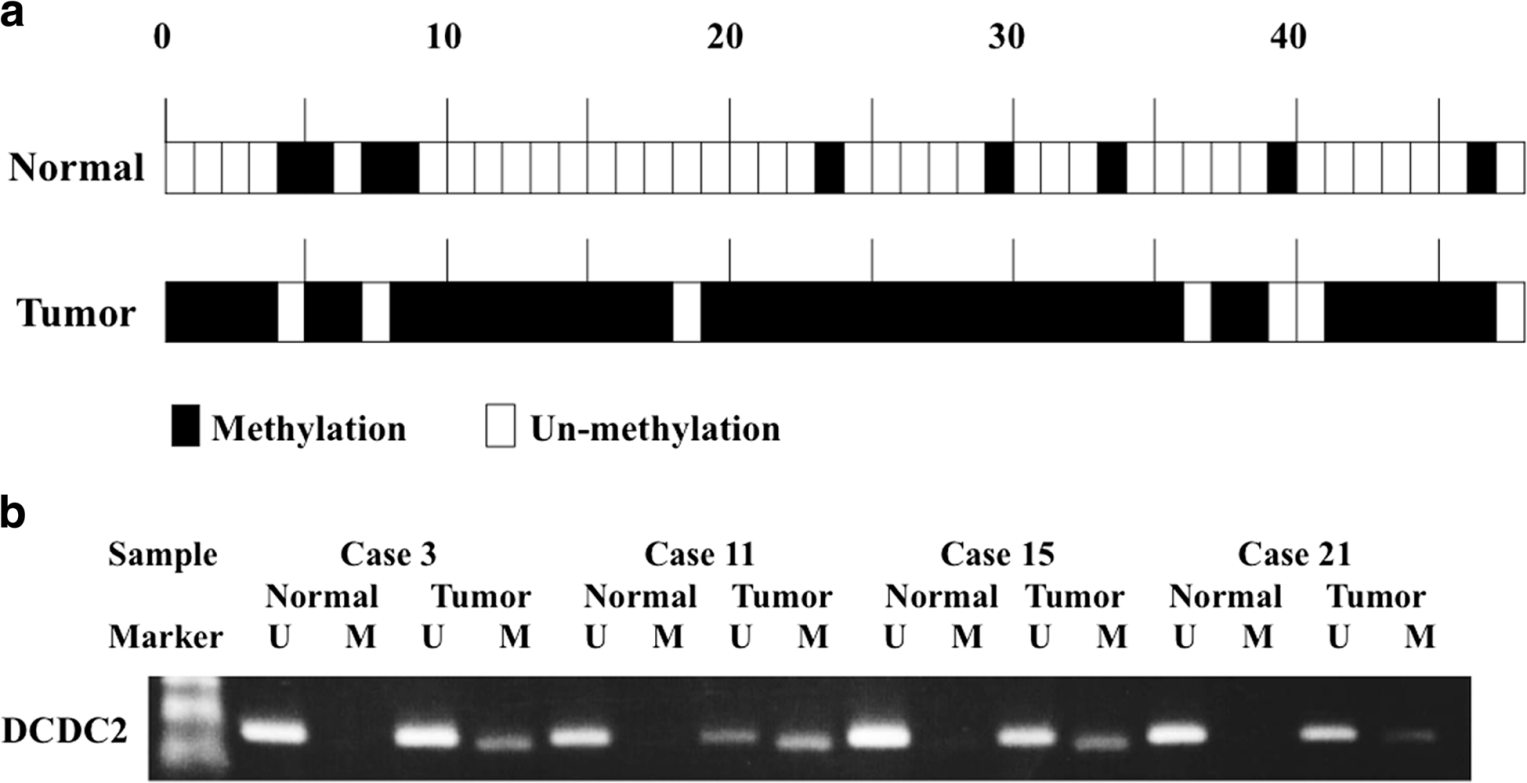
**Results of MSP in 48 HCC cases. (a)** Methylation status in 48 primary HCC samples. Forty-one of 48 (85.4%) cancer tissues showed hypermethylation of *DCDC2*, while only 9 of 48 (18.7%) cases showed hypermethylation in adjacent normal tissues. **(b)** Four representative cases showing hypermethylation of the promoter region of *DCDC2* in tumor tissues without methylation in normal tissues.

Overall, 41 of the 48 (85.4%) tumor samples displayed *DCDC2* promoter hypermethylation, whereas only 9 of 48 samples showed hypermethylation in the normal samples (Figure 4a). Thus, hypermethylation of *DCDC2* was significantly more frequent in tumor tissues (*P* < 0.001). Four representative cases of MSP and UN-MSP status are shown in Figure 4b.

### Real-time quantitative RT-PCR analysis of 48 HCC patients

**Figure 5:**
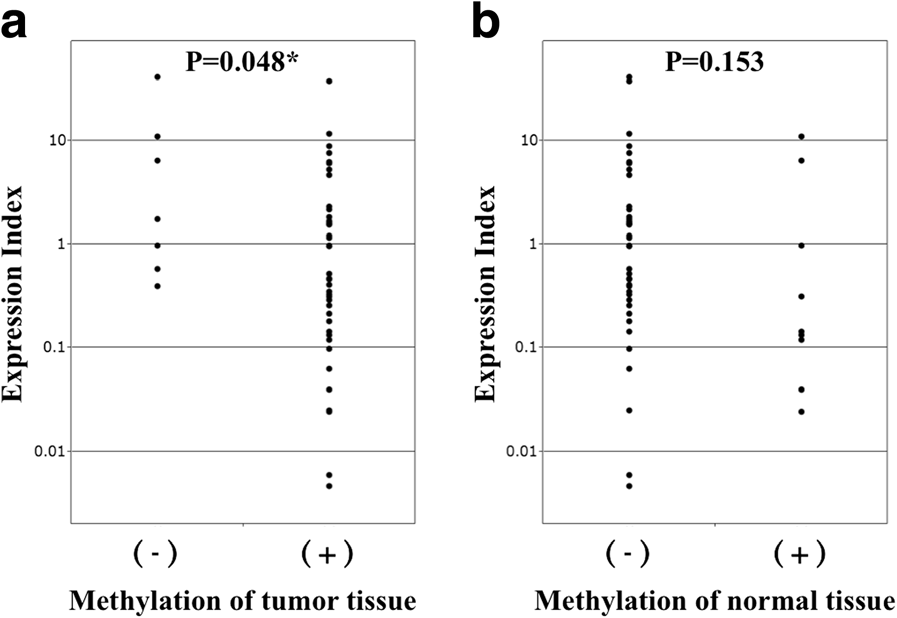
**Results of quantitative RT-PCR in 48 HCC cases. (a)** Expression levels of *DCDC2* mRNA examined by RT-PCR in 48 cases. The expression index [(*DCDC2*-tumor) × (GAPDH-normal)/(*DCDC2*-normal) × (GAPDH-tumor)] was calculated for all 48 cases. Expression index in methylated cases were significantly lower than unmethylated cases (*P* = 0.048). **(b)** Methylation in normal tissue did not show significant difference in expression index (*P* = 0.153).

We also examined the expression levels of *DCDC2* mRNA by real-time RT-PCR in the 48 analyzed cases, calculated as the value of *DCDC2* mRNA expression divided by that of *GAPDH* for each sample. The *DCDC2* expression index was calculated as the value of the tumor tissue expression level divided by that of the expression level of the adjacent normal tissue. With regard to the correlation between methylation status and expression index, hypermethylated cancerous tissues had significantly lower *DCDC2* expression index than other tissue examined (*P* = 0.048; Figure 5a). However, methylation in normal tissue did not show significant difference in expression index (*P* = 0.153; Figure 5b).

### Western blotting

**Figure 6:**
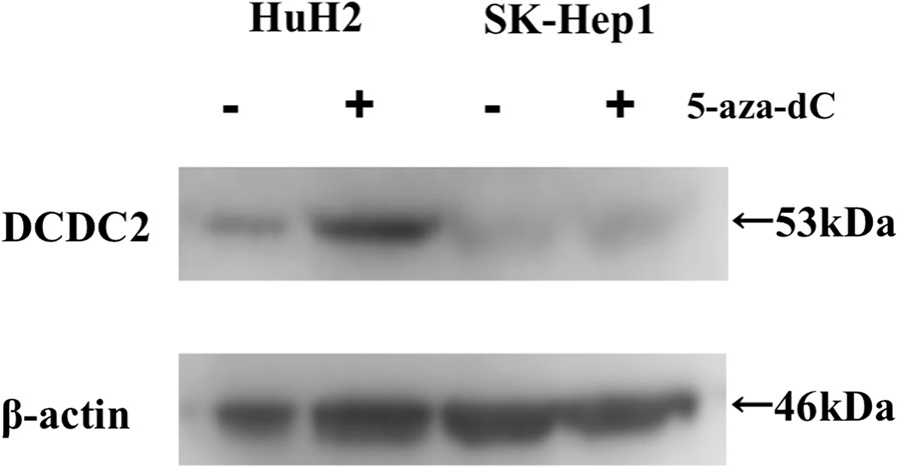
Western blotting analysis showed reactivation of DCDC2 protein by 5-aza-dC treatment in HuH2 cells that were completely methylated, whereas reactivation was not observed in SK-Hep1 cells that were completely unmethylated.

Evaluation by western blotting confirmed DCDC2 protein expression after 5-aza-dC treatment in HuH2 and SK-Hep1 cells was consistent with that of RT-PCR. The expression of *DCDC2* in the cells was also reactivated by the treatment in HuH2 cells that were completely methylated (Figure 6).

### Immunohistochemical staining of *DCDC2*

**Figure 7:**
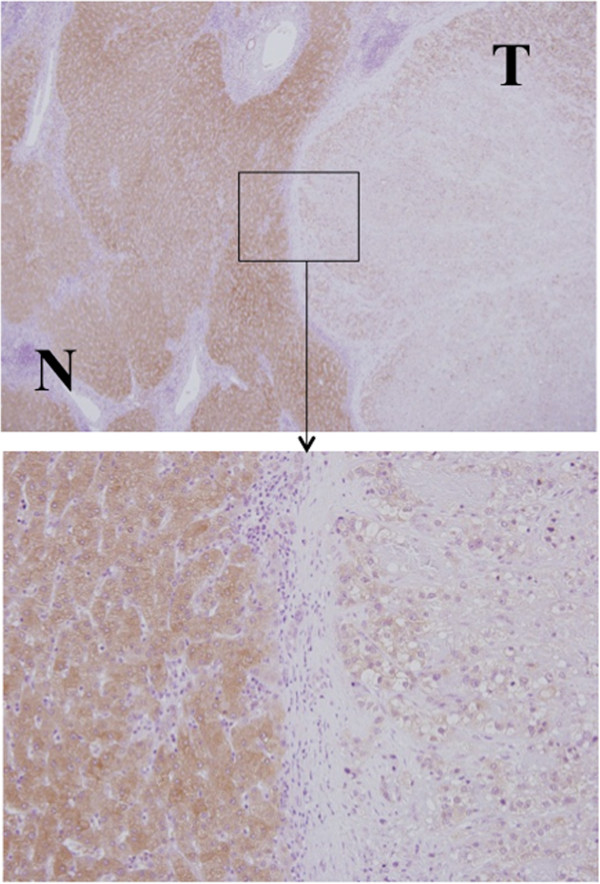
**Representative finding of immunohistochemical staining of DCDC2 in a resected sample.** Strong staining was observed in the cytoplasm of non-cancerous cells, whereas weak staining was present in tumor cells (upper picture: magnification 40×, lower picture: magnification 200×).

In the 24 (63.1%) of 38 cases that underwent immunohistochemical staining, the cancerous components showed reduced DCDC2 protein expression compared with adjacent non-cancerous tissue. In 18 of 31 methylated cases, and in six of seven unmethylated cases, the cancerous tissues showed downregulated DCDC2, and there was no significant relationship between methylation status and DCDC2 protein expression, suggesting that there could be other silencing mechanisms involved in HCC (Figure 7).

### Correlation between promoter hypermethylation status of *DCDC2* gene and clinicopathological characteristics in 48 HCC patients

**Figure 8:**
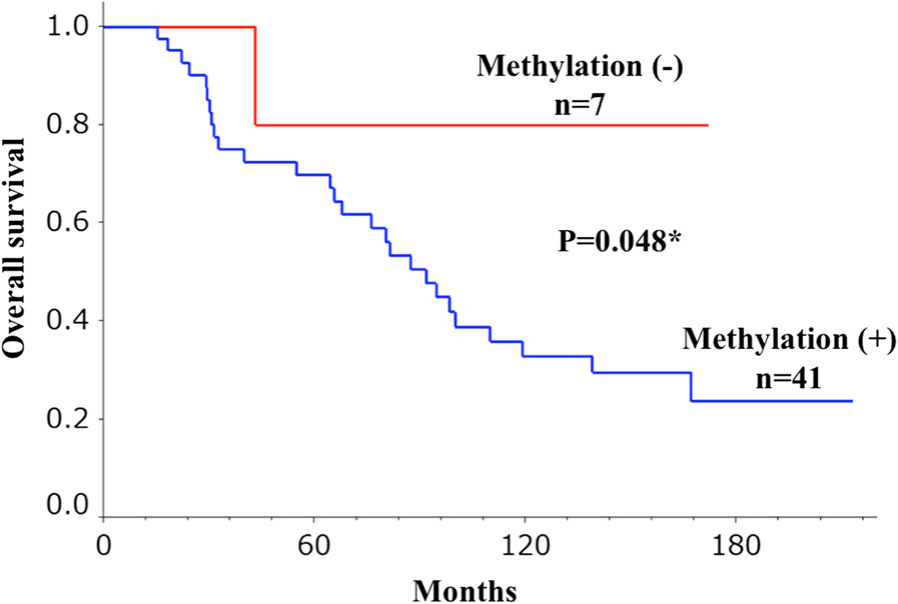
**Overall survival stratified by methylation status of *DCDC2*.** Methylated cases of tumor tissues were significantly correlated with a worse prognosis compared with that of unmethylated cases (*P* = 0.048).

We analyzed the correlation between the hypermethylation status of *DCDC2* and clinicopathological features of the 48 HCC patients. Whereas no notable association between the methylation status and clinicopathological variables was detected (data not shown), the methylated cases showed poorer prognosis of overall survival than the unmethylated cases (*P* = 0.048; Figure 8).

## Discussion

Recent studies have investigated the relationship between carcinogenesis and DNA methylation in different cancer types [28-30]. Methylation in a number of genes in HCC has also been investigated worldwide [31-34]. However, it is unknown whether methylated genes in HCCs are associated with gene expression, or prognosis of the patients.

To detect new cancer-related genes that enable prediction of the prognosis of patients who undergo hepatectomy for HCC, we developed a double combination array analysis consisting of expression array and SNP array analysis, and have reported several genes associated with hepatocarcinogenesis [12-17]. Our experiment proves that these genes were hypermethylated in HCC tumor tissues, resulting in decreased expression and poorer prognosis, and we realized the double combination array analysis was an efficient procedure to identify new cancer-related genes via an epigenetic mechanism. However, this procedure required validation in HCC specimens on the basis that the downregulation of these genes occurred by methylation of promoter regions. To ensure the involvement of gene methylation, we developed a triple combination array analysis that consists of expression array, SNP array, and methylation array analysis, and reported a new tumor suppressor gene using this procedure [18].

In the current study, we identified *DCDC2* as a candidate tumor suppressor gene in HCC using triple combination array analysis. The promoter region of this gene was hypermethylated in many cancer tissues but only in a few normal tissues. The expression of *DCDC2* in tumor tissues was decreased in methylated cases (*P* = 0.048). The overall survival of the patients with *DCDC2* methylation was significantly worse than those without methylation (*P* = 0.048).

*DCDC2* has been reported as a gene related with dyslexia [21-24]. DCDC2 protein is considered to have important roles in neural migration and construction of microtubules [19-21]. Massinen et al. showed downregulation of DCDC2 expression enhanced Wnt signaling, which is important in neuronal development [35]. Moreover, it is known that aberrant activation of the Wnt pathway is associated with human malignancies, including HCC [36,37]. Therefore, it could be hypothesized that methylation of *DCDC2* downregulates the expression of its protein product to cause activation of the Wnt pathway and worsen the prognosis of HCC patients. To support this hypothesis, various studies investigated secreted frizzled-related protein 1 (*SFRP1)* in HCC [38-41], and Kaur P et al. indicated *SFRP1* expression was downregulated by methylation resulting in activation of the Wnt pathway and contributing to increased HCC cell growth and proliferation [41]. Therefore, *DCDC2* might play a role in HCC in similar way to *SFRP1*.

One of the limitations of this method is that we can obtain array information from only one pair of resected specimens at a time. However, we identified *DCDC2* by triple combination array analysis. Thus, we investigated this gene in 48 resected HCC specimens and proved the impact of methylation in cancer tissues. The relevance of *DCDC2* in the tumorigenesis of HCC could therefore be considered as universal. Another limitation is that the results of immunohistochemical staining and DNA methylation status did not show significant association. We consider that the methylation is not the only mechanism that regulates the protein expression. Other mechanisms such as histone deacetylation or post-transcriptional regulation by microRNAs might play a role in regulation of DCDC2 protein expression [42,43]. However, our results showed the contribution of methylation in mRNA expression and prognosis after surgery.

Taken together, the methylation of *DCDC2* could be a prognostic marker after surgical resection of HCC. Furthermore, decitabine has become a therapeutic agent for patients with myelodysplastic syndrome (MDS) by DNA hypomethylation [44]. It is considered that p15 and other methylated genes may be therapeutic targets of the DNA methylation-inhibitory activity of decitabine in MDS [45]. In the future, it might be applied in the clinical setting for HCC patients who have methylated *DCDC2* in their tumor tissue.

## Conclusions

In conclusion, our triple combination array analysis detected *DCDC2* as a candidate tumor suppressor gene in HCC. Additional investigations of the function of this gene in carcinogenesis are required to confirm this gene as a bona fide tumor suppressor. According to our clinical data from 48 HCC specimens, the extent of promoter hypermethylation for this gene correlated with overall survival. Further studies will be required to evaluate the effect of *DCDC2* re-expression in HCC cells by a methylation inhibitor. If re-expression with such an agent can inhibit tumor growth, this may represent a key line of therapy for advanced HCC tumors.

## Abbreviations

DCDC2: Doublecortin domain-containing 2; HCC: Hepatocellular carcinoma; SNP: Single nucleotide polymorphism; RT-PCR: Reverse transcription-polymerase chain reaction; MSP: Methylation specific polymerase chain reaction; LOH: Loss of heterozygosity; HT: HCC tissue; NT: Normal tissue; 5-aza-dC: 5-Aza-2′-deoxycytidine; SFRP1: Secreted frizzled-related protein 1; MDS: Myelodysplastic syndrome.

## Competing interests

The authors declare that they have no competing interests.

## Authors’ contributions

YI: Analyzing data, experimental work, and drafting article. SN: Conception, design, experimental work, and acquiring data from array analysis. MH: Experimental work. MH: Analyzing data and experimental work. MK: Experimental work. YN: Experimental work. ST: Sample collection. HS: Sample collection. TF: Sample collection SY: Sample collection. YK: Sample collection. All authors read and approved the final manuscript.
